# Concurrent Dual Contrast for Cellular Magnetic Resonance Imaging Using Gadolinium Oxide and Iron Oxide Nanoparticles

**DOI:** 10.1155/2012/230942

**Published:** 2012-08-02

**Authors:** Yasir Loai, Tameshwar Ganesh, Hai-Ling Margaret Cheng

**Affiliations:** ^1^Physiology and Experimental Medicine, The Research Institute, The Hospital for Sick Children, Toronto, ON, Canada M5G 1X8; ^2^Department of Medical Biophysics, Faculty of Medicine, University of Toronto, Toronto, ON, Canada

## Abstract

*Rationale and Objectives*. Concurrent visualization of differential targets in cellular and molecular imaging is valuable for resolving processes spatially and temporally, as in monitoring different cell subtypes. The purpose of this study was to demonstrate concurrent, dual (positive and negative) contrast visualization on magnetic resonance imaging (MRI) of two colocalized cell populations labeled with Gadolinium “Gd” oxide and iron “Fe” oxide nanoparticles. *Materials and Methods*. Human aortic endothelial cells (EC) and smooth muscle cells (SMC) were labeled with various concentrations of Gd oxide and Fe oxide, respectively. MRI on single- or mixed-cell samples was performed at 7 tesla. Proper cell phenotype expressions, cell uptake of contrast agents, and the effect of labeling on cell viability and proliferation were also determined. *Results*. Both contrast agents were efficiently taken up by cells, with viability and proliferation largely unaffected. On MRI, the positive contrast associated with Gd oxide-labeled EC and negative contrast associated with Fe oxide-labeled SMC discriminated the presence of each cell type, whether it existed alone or colocalized in a mixed-cell sample. *Conclusion*. It is feasible to use Gd oxide and Fe oxide for dual contrast and concurrent discrimination of two colocalized cell populations on MRI at 7 tesla.

## 1. Introduction

Contrast agents play a critical role in cellular and molecular magnetic resonance imaging (MRI), as they enable sensitive and clear visualization of cellular and physiological processes. The different contrast agents available for labeling cells share in common their ability to enhance signal contrast and have facilitated cell-tracking studies in stem cell transplantation, neurodegenerative disorders and stroke, atherosclerosis, and cancer research [1–4]. Superparamagnetic iron oxide nanoparticles are by far the most common, owing to their strong negative *T*_2_-weighted contrast, and have been widely used for tracking different cell types in various organs, including lymphocytes, progenitor cells, and embryonic cells [5–9]. Less common are paramagnetic gadolinium (Gd-) based agents, which exploit positive *T*_1_-weighted contrast changes. These have been used in their chelated form for tracking stem cells in animal models of hemorrhagic transformation [10] and angiogenesis [11] and, more recently, in a nanoparticle formulation for improved cell uptake and retention [12]. However, in virtually all of the reported cellular MRI applications, only one cell population could be labeled and tracked.

 The ability to track two or more cell populations is potentially very powerful as it confers the power to monitor cell interactions and the development of complex tissues and organs. Such capability would require the use of different contrast agents for labeling different targets. Gilad et al. [13] used iron oxide and manganese oxide nanoparticles to distinguish labeled glioma cells injected into contralateral sides of the rat brain. The same group also demonstrated that iron oxide and a chemical exchange saturation transfer (CEST) agent in solution could be imaged simultaneously and distinguished from each other [14]. CEST agents are attractive as a “tunable” agent, since they are detected only when a saturation pulse is applied at a specific frequency [15]. However, unlike iron oxides and Gd compounds, the sensitivity of CEST agents is much lower and their production and design are currently restricted to research laboratories. The combination of iron oxide and Gd-based contrast agents, therefore, provides an attractive and practical platform for developing dual contrast visualization and tracking of differential targets.

In this study, a Gd oxide nanoparticle (Gado *CELL* Track, BioPAL) and an iron oxide nanoparticle (Molday ION Rhodamine, BioPAL, Worcester, MA) were investigated for labeling different cell populations and using dual (positive and negative) contrast for their concurrent distinction on MRI at 7 Tesla. The Gd oxide is a novel 50 nm Gd colloid analogous to iron oxides and is much larger than conventional Gd chelates. The contrast agents were used to separately label normal human aortic endothelial and smooth muscle cells, respectively, chosen for their use in vascularization of engineered tissues. MRI measurements of *T*_1_- and *T*_2_-relaxation times and assays on cell viability and proliferation were performed to determine the concentrations at which the two contrast agents can be used concurrently. Results demonstrate that the different cell types could be distinguished simultaneously in a mixed cell population. To our knowledge, this is the first proof-of-principle demonstration of concurrent dual contrast using iron oxide and Gd oxide nanoparticles for labeling and visualizing different colocalized cell targets.

## 2. Materials and Methods

### 2.1. Cell Lines and Cell Culture

Normal human aortic endothelial cells (EC) (HAoEC; FC-0014) and smooth muscle cells (SMC) (HAoSMC; FC-0015) were obtained from Lifeline Cell Technology (Walkersville, MD). They were maintained in basal medium complete with appropriate supplements, VascuLife EnGS Medium Complete (LL-0002), and VascuLife SMC Complete (LL-0014), and 1% penicillin/streptomycin. Fresh medium (13 mL) was added every two days, and cells were passaged 1 : 3 at 80% confluence in T75 flasks (BD Biosciences, Mississauga, ON, Canada) and incubated at 37°C, 5% CO_2_. Before passaging, cells were washed twice with 5 mL of sterile phosphate-buffered saline (PBS) and incubated with 3 mL 0.25% Trypsin/0.1 mM EDTA for 3 minutes. Then, 7 mL of medium with fetal bovine serum was added to the cells. The entire 10 mL was collected in 15 mL tubes and spun at 1300 rpm for 5 minutes. The liquid above the cell pellet was aspirated, and cells were resuspended in fresh medium and passaged. Cells were passaged and maintained according to company instructions.

### 2.2. Contrast Agent and Cell Labeling

Two contrast agents were employed in this study for cell labeling, both purchased from BioPAL, Inc. (Worcester, MA). The first was Molday ION Rhodamine (CL-50Q02-6A-50), a superparamagnetic iron oxide (SPIO) nanoparticle having a colloidal size of 50 nm and labeled with rhodamine B, a fluorescent dye, to allow visualization on both MRI and fluorescence. The second was Gado *CELL* Track (-) (CL-50P02-6), a Gd oxide (Gd_2_O_3_) nanoparticle having a colloidal size of 50 nm. To enhance cell uptake, the Gd_2_O_3_ nanoparticle was mixed with poly-L-lysine (PLL) (CL-00-01, BioPAL). Small volumes of Gd_2_O_3_ and PLL (2.5 : 1 ratio) were incubated in sterilized 1.5 mL eppendorf tubes at room temperature (RT) for one hour to allow complete solubilization.

EC and SMC were grown in 12-well plates (BD Falcon, Mississauga, ON, Canada) for 2 days in a 37°C, 5% CO_2_ humidified incubator. When 70–80% confluence was reached, 1 mL of fresh media was added. EC were incubated with Gd_2_O_3_ and SMC were incubated with SPIO, returning to the incubator for 24 hours. The following concentrations were prepared for the incubation medium: 0, 0.002, 0.02, 0.1, and 0.2 mM of Gd; 0, 0.0036, 0.009, 0.018, and 0.036 mM of iron (Fe). Cells were washed 3 times with sterile 1x PBS to remove excess contrast agents. Fresh supplemented medium was then given to the cells (1 mL/well). The medium was changed every 2 to 3 days with subsequent washing, with the last wash carried out prior to MRI.

### 2.3. Determination of Gd and Iron Content in Cells

Samples were assayed for Gd and Fe content using inductively coupled plasma-atomic emission spectroscopy (ICP-AES) at the ANALEST facility (Toronto, ON, Canada). The analysis was performed on a Perkin Elmer spectrometer (Model Optima 7300DV ICP AEOS) and certified against independent sources traceable to the National Institute of Standards and Technology Standard Reference Material. The detection threshold was 0.01 *μ*g/mL for both Gd and Fe. The cellular elemental concentration was determined by dividing the total content by the number of cells.

### 2.4. Immunofluorescence

Cell phenotype was confirmed by visualizing the expression of EC-specific Von Willebrand factor and *α*-smooth muscle actin for SMC using single and double immunofluorescence. Cells were grown on sterile coverslips for 3 days to allow for adhesion and proliferation. Samples were then fixed in 5% formalin in PBS for 15 minutes at RT. After washing twice with 1x PBS, cells were permeabilized with ice-cold methanol for 10 minutes at −20°C to target internalized protein markers. Excess methanol was washed off twice with 1xPBS and blocked with 4% bovine serum albumin/PBST (PBS + 0.05% Tween) at RT for 1 hour. For primary antibodies, EC were incubated with mouse monoclonal Von Willebrand factor IgG1 antibody (1 : 300; Abcam, ab68545, Cambridge, MA) for 2 hours at RT. Under the same conditions, SMC were incubated with monoclonal antiactin, *α*-smooth muscle-FITC mouse antibody (1 : 200; Sigma, F3777, St. Louis, MO). Primary antibodies were washed 5 times (5 minutes/wash) with 1x  PBST. For the secondary antibody, EC were incubated with polyclonal goat antimouse IgG-H&L (Texas Red) (1 : 400; Abcam, ab6787) for 1 hour at RT. Excess antibody was washed off 5 times with 1x PBS (5 minutes/wash) and counterstained with VECTASHIELD mounting medium with DAPI for 1 minute (Vector Laboratories, H-1200, Burlingame, CA) and mounted onto slides.

Presence of SPIO in cells was additionally confirmed through immunofluorescence of the rhodamine B dye tagged to the iron oxide. Cultured EC and SMC were seeded onto coverslips (10,000 cells/coverslip) and left to adhere for 1 day, then incubated with SPIO at 0.0036 mM for 24 hours. Afterwards, cells were washed 3 times with 1x PBS, incubated with fresh medium, and left to grow for 1, 3, and 7 days at 37°C in a 5% CO_2_ incubator. Cells were then gently washed 3 times with 1x PBS, counterstained with VECTASHIELD mounting medium with DAPI for 1 minute, and mounted onto slides.

### 2.5. Cell Viability and Proliferation Assays

To assess cell viability and proliferation, 96-well assay plates containing EC or SMC (~8,000 cells in 250 *μ*l of culture medium per well) were set up. Following a 24-hour period of cell adhesion onto the well plates, Gd_2_O_3_ was added to EC and SPIO was added to SMC using the same concentrations as in the 12-well plate preparations for MRI. Cells were incubated with contrast agent for 24 hours, washed 3 times with 1x PBS, and either assayed immediately (1 day group) or incubated in 250 *μ*l fresh culture medium for an additional 2 days (3 day group) or 6 days (7 day group). The medium was replaced every 48 hours. Fresh medium (100 *μ*l) was replenished prior to performing the following assays.

CellTiter-Blue Cell Viability Assay (Promega, G8080, Madison, WI) was used to assess cell cytotoxicity following incubation. 20 *μ*l of CellTiter-Blue reagent was added to each well, shaken for 10 seconds, and incubated at 37°C, 5% CO_2_ for 2 hours. Fluorescence was recorded at 560/590 nm (Spectra MAX Gemini Microplate Spectrofluorometer).

Cell Titer 96 Aqueous One Solution Cell Proliferation Assay (MTS) (Promega, G3582) was used to determine the number of viable cells undergoing proliferation. 20 ul of combined MTS/PBS solution was dispensed into each well. Cells were incubated for 2 hours at 37°C, 5% CO_2_. Absorbance was recorded at 490 nm.

For all assays, samples were set up as triplicates. Blank wells (media with reagent alone) and controls (media with unlabeled cells) were included. Fluorescence versus concentration of test samples was plotted using GraphPad Prism (LaJolla, CA).

### 2.6. MR Imaging

MR imaging was performed on a 7 tesla preclinical magnet (BioSpec 70/30, Bruker, Ettlingen, Germany) using Bruker commercial hardware, namely, a B-GA12 gradient coil, a 7.2 cm cylindrical linear transmit coil, and a 4-channel murine phased-array receiver coil. Prior to MRI, cells were prepared as follows. From the 12-well plates used to cultivate cells for MRI, approximately 250,000 cells per well were harvested using 100 *μ*L 0.25% Trypsin/0.1 mM EDTA and subsequent 100 *μ*L 10% fetal bovine serum medium. Cells were transferred to 200 *μ*L pipette tips, with bottoms sealed through melting and the tips capped with PCR tube caps. Two pipette tips were placed in a larger 15 mL tube, which was spun for 10 minutes at 1500 rpm to create a cell pellet. Subsequently, the old medium was aspirated from the tips, and 200 *μ*L of fresh medium was added on top of the cells. There were approximately 250,000 cells per pipette tip. Mixtures of EC and SMC were prepared at a ratio of approximately 55 (EC) : 45 (SMC).

Cell pellets in the pipette tips were kept on ice during the transfer to MRI. The tips were then secured firmly in an upright position in plasticine and placed on the phased-array coil. Quantitative *T*_1_-relaxation times were measured using a 2D saturation-recovery rapid acquisition with relaxation enhancement (RARE) sequence [16]: RARE factor = 2, repetition time (*T*_*R*_) = [5000, 3000, 2000, 1500, 1000, 750, 500, 250, 150, 125 ms], echo time (*T*_*E*_) = [7.43, 22.3, 37.1, 52.0, 66.8, 81.7, 96.6, 111.4 ms], field-of-view = 2.56 cm, slice thickness = 4 mm, in-plane resolution = 100 × 100 um, rf excitation bandwidth = 2000 Hz, receive bandwidth = 81.5 kHz, and scan time = 22 min. Quantitative *T*_2_-relaxation times were measured using a spin-echo Carr-Purcell-Meiboom-Gill (CPMG) sequence [17]: *T*_*R*_ = 4000 ms, 128 echoes with *T*_*E*_ *s* = [4.19, 8.38,…, 536.5 ms], slice thickness = 5 mm, in-plane resolution = 200 × 200 um, number of averages = 2, rf excitation bandwidth = 7500 Hz, receive bandwidth = 75 kHz, and scan time = 13 min. Relaxometry measurements were performed at ambient temperature.

### 2.7. Data Analysis

MR raw data were transferred to an independent workstation for quantitative data analysis. All data processing was performed using in-house software developed in MATLAB (v.7.8) (MathWorks, Natick, MA). Quantitative maps of *T*_1_-relaxation times were computed by nonlinear least squares fitting to the signal intensity on the saturation-recovery curve at the minimum echo time. Quantitative *T*_2_-relaxation times were computed by fitting signal intensity to a monoexponential decay function added to a constant offset to account for noise. All data were expressed as mean value ± standard deviation. All significance testing was based on the Student's *t*-test, with a probability value *P* < 0.05 considered significant.

## 3. Results

### 3.1. Endothelial Cell and Smooth Muscle Cell Cultures

**Figure 1:**
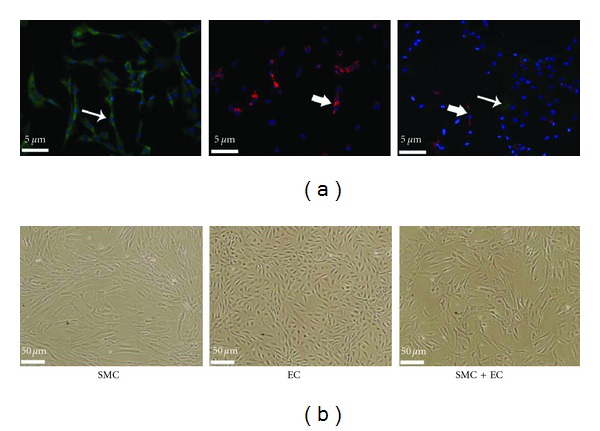
Normal human SMC and aortic EC (passage 2) cultured separately and together (SMC + EC). (a) Immunofluorescence expression of Von Willebrand factor (previously known as factor VIII) for EC and *α*-smooth muscle actin for SMC. Arrows indicate Von Willebrand factor (red) and *α*-smooth muscle actin (green) staining. DAPI (blue) staining refers to fixed cell nuclei (20x magnification). (b) Phase contrast microscopy (10x magnification).

Prior to cell labeling, proper cell morphology and phenotype were confirmed for cultured human aortic endothelial cells (EC) and smooth muscle cells (SMC). Immunofluorescence identified the expression of *α*-smooth muscle actin for SMC and Von Willebrand factor (previously known as Factor VIII) for EC (Figure 1(a)). The morphological appearances of cells are shown in the phase contrast microscope images of Figure 1(b).

### 3.2. Labeling Endothelial Cells with Gadolinium Oxide

Endothelial cells were labeled with a gadolinium oxide (Gd_2_O_3_) nanoparticle at various concentrations for an incubation interval of 24 hours. The mean cellular uptake of Gd_2_O_3_, expressed as pg Gd/cell, was assessed by ICP-AES and summarized in Table 1. Contrast agent uptake was linear with the concentration of Gd_2_O_3_ in the incubation medium (*r* = 0.995, *P* < 1*e* − 4), with approximately onehalf of the Gd_2_O_3_ in solution internalized by cells.

**Figure 2:**
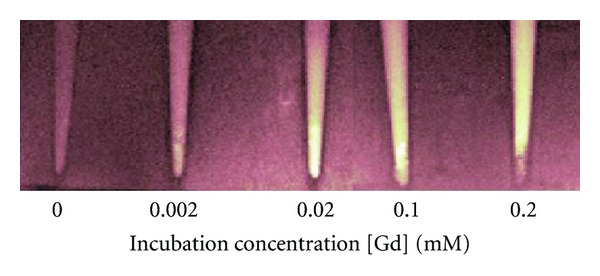
EC pellets labeled with Gd_2_O_3_ on *T*_1_-weighted MRI at 7 Tesla. Cells were incubated in medium with different [Gd] concentrations and imaged at 1 day after labeling. Scan parameters were *T*_*R*_/*T*_*E*_ = 1000/7.4 ms.

**Figure 3:**
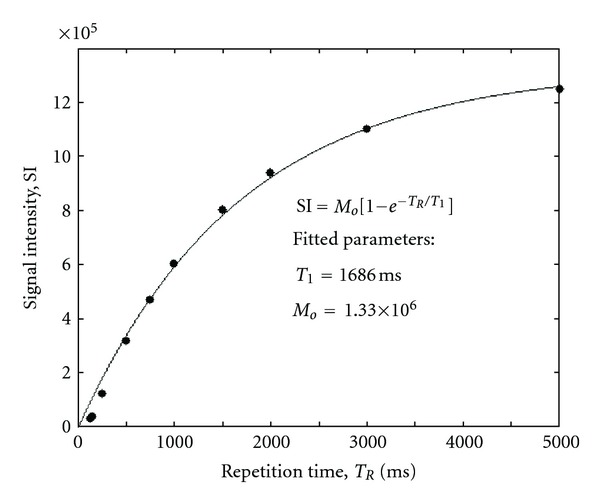
Example of *T*_1_-relaxation measurements (filled circles) in EC pellets labeled with Gd_2_O_3_ in a 0.02 mM [Gd] solution. Regression fit (solid line) to exponential recovery equation and fitted parameters are shown.

The appearance of Gd_2_O_3_-labeled EC pellets on MRI at 7 Tesla is illustrated in Figure 2. All labeled cell pellets could be identified on the basis of altered signal contrast compared to the overlying medium. Unlabeled cells, however, were not distinguished. Increased signal intensity on *T*_1_-weighted images was achieved in cells incubated at low concentrations of Gd_2_O_3_, which arises from enhanced *T*_1_-relaxation (i.e., evident as lower *T*_1_ relaxation times) with increasing concentration. Measured relaxation times in labeled EC for all incubation concentrations are provided in Table 2.  Figure 3 illustrates relaxation data in a labeled EC pellet from which *T*_1_ is estimated. Note also in Figure 2 that at a concentration of 0.1 mM competing effects began to offset *T*_1_ enhancement, and at 0.2 mM signal loss dominated. This competing effect arose from a concurrent *T*_2_ reduction (see Table 2) that was especially pronounced at concentrations of 0.1 mM and higher. It is important to note that although positive contrast relies primarily on a lower *T*_1_, whether or not we can see the *T*_1_-dependent signal increase also depends on *T*_2_, which, if very low, can obliterate *T*_1_-induced enhancement. Despite the reversal of MR signal contrast, higher incubation concentrations did not exert deleterious effects on cell function. Cell viability and proliferation were unaffected relative to unlabeled cells for the Gd_2_O_3_ concentrations tested, except for a decreased proliferative capacity noted at 0.2 mM (*P* < 0.05).

### 3.3. Labeling Smooth Muscle Cells with Iron Oxide

Smooth muscle cells were labeled with an iron oxide (SPIO) nanoparticle at various concentrations for an incubation interval of 24 hours. The mean cellular uptake of SPIO, expressed as pg Fe/cell, was assessed by ICP-AES and summarized in Table 1. Contrast agent uptake was linear with the concentration of SPIO in the incubation medium (*r* = 0.9997, *P* < 1*e* − 6), with approximately 87% of the SPIO in solution internalized by cells.

**Figure 4:**
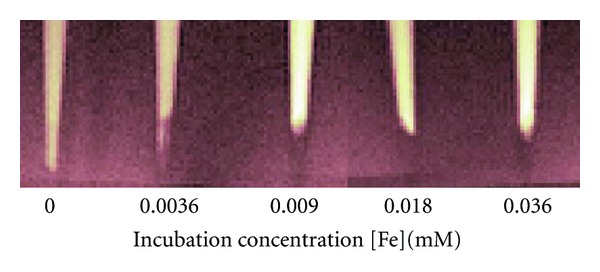
SMC pellets labeled with SPIO on *T*_2_-weighted MRI at 7 Tesla. Cells were incubated in medium with different [Fe] concentrations and imaged at 1 day after labeling. Scan parameters were *T*_*R*_/*T*_*E*_ = 4000/23.8 ms.

**Figure 5:**
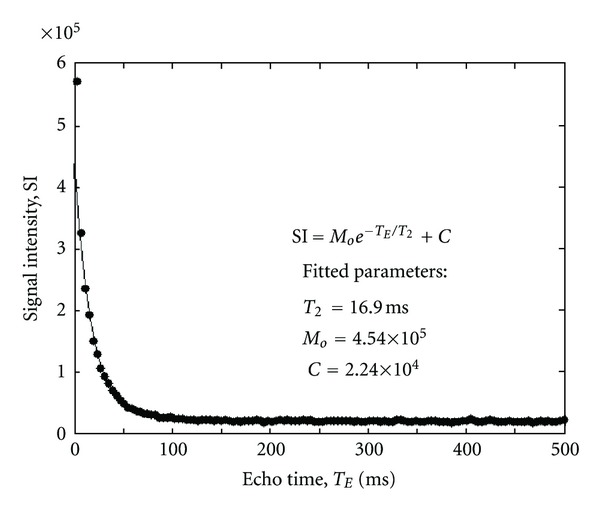
Example of *T*_2_-relaxation measurements (filled circles) in SMC pellets labeled with SPIO in a 0.0036 mM [Fe] solution. Regression fit (solid line) to exponential decay equation and fitted parameters are shown.

The appearance of SPIO-labeled SMC pellets on MRI at 7 Tesla is illustrated in Figure 4. All labeled cell pellets could be identified on the basis of negative signal contrast compared to the overlying medium. Unlabeled SMC were not distinguished, similar to unlabeled EC shown in Figure 2. Decreased signal intensity on *T*_2_-weighted images arose primarily from a significantly lowered *T*_2_-relaxation time, which was achieved at an incubation concentration of 0.0036 mM. Measured relaxation times in labeled SMC for all incubation concentrations are provided in Table 3.  Figure 5 illustrates relaxation data in a SMC pellet from which *T*_2_ is estimated. With increasing concentration, further reductions in *T*_2_ were relatively minor compared to *T*_1_ lowering. Assays on cell function showed that viability and proliferation were both unaffected relative to unlabeled cells for all SPIO concentrations tested (see Table 3).

### 3.4. Concurrent Dual Contrast of Labeled Endothelial and Smooth Muscle Cells

**Figure 6:**
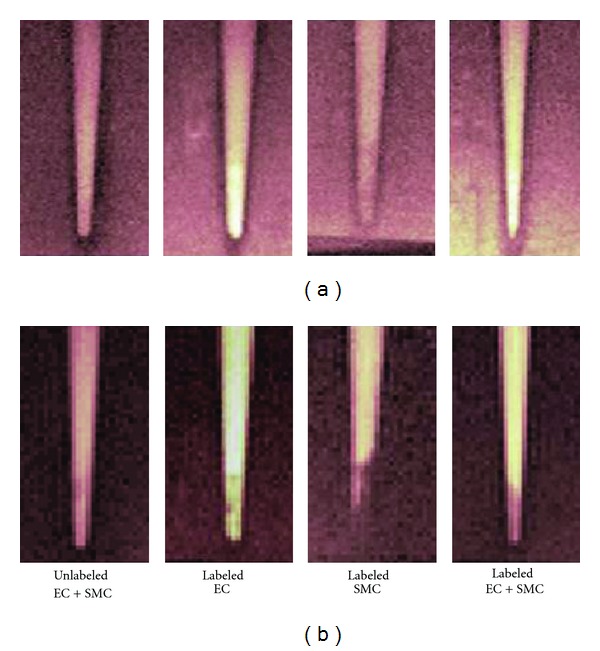
(a) *T*_1_-weighted and (b) *T*_2_-weighted MR images of labeled cell pellets at 7 Tesla. EC were labeled with Gd_2_O_3_. SMC were labeled with SPIO. Imaging was performed at 1 day post-labeling. Scan parameters were *T*_*R*_/*T*_*E*_ = 1000/7.4 ms for *T*_1_-weighted scans, *T*_*R*_/*T*_*E*_ = 4000/41.9 ms for *T*_2_-weighted scans.

Ideally, one form of image contrast (e.g., positive *T*_1_) would be used to identify one cell type, whether it exists alone or in a mixture with another cell type; and the absence of image contrast would indicate the absence of that cell type. Similarly, the second form of image contrast (e.g., negative *T*_2_) would be used to identify specifically the other cell type. To achieve the desired simultaneous and distinct cell identification, appropriate contrast incubation concentrations must be selected to achieve concurrent dual contrast of labeled cells. For this purpose, low concentrations of SPIO were used to generate a significant negative *T*_2_ contrast while maintaining good spatial definition. Low concentrations of Gd_2_O_3_ were also used to attain a significant *T*_1_ enhancement without incurring *T*_2_ decreases of a magnitude used for identifying the presence of SPIO.  Figure 6 illustrates the MRI appearance of cell pellets containing Gd_2_O_3_-labeled EC and SPIO-labeled SMC, both separately and in combination. Positive contrast on *T*_1_-weighted images (Figure 6(a)) identifies correctly the presence of labeled EC, alone or mixed with labeled SMC; labeled SMC alone and unlabeled cells do not generate positive contrast. Negative contrast on *T*_2_-weighted images (Figure 6(b)) also correctly identifies the presence of labeled SMC, alone or mixed with labeled EC; labeled EC alone generates a comparatively weak negative signal.

**Figure 7:**
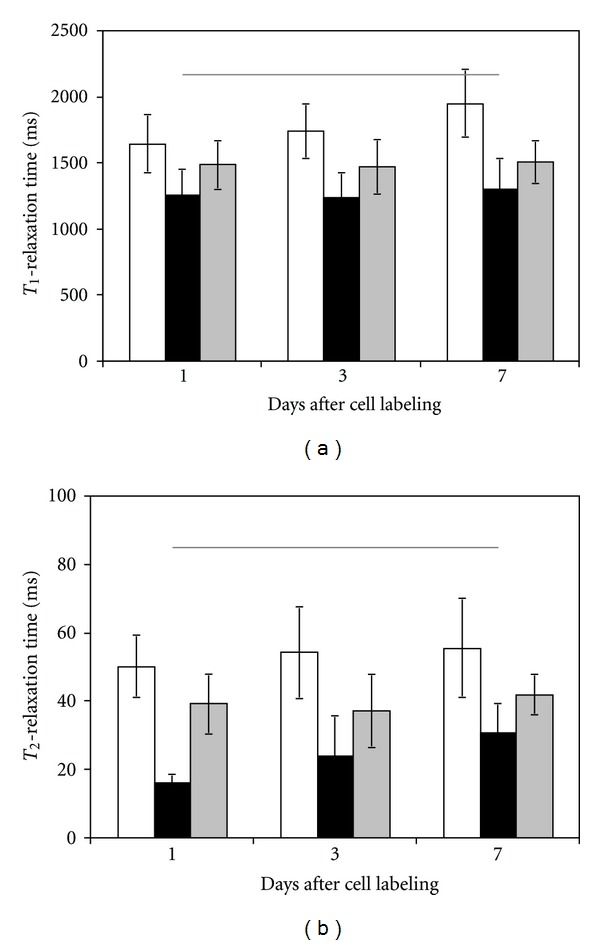
*T*_1_- and *T*_2_-relaxation times in labeled cell pellets over 7 days after labeling. Gd_2_O_3_-labeled EC (white), SPIO-labeled SMC (black), and mixed labeled EC and SMC (gray). Horizontal lines indicate relaxation times in unlabeled cells.

**Figure 8:**
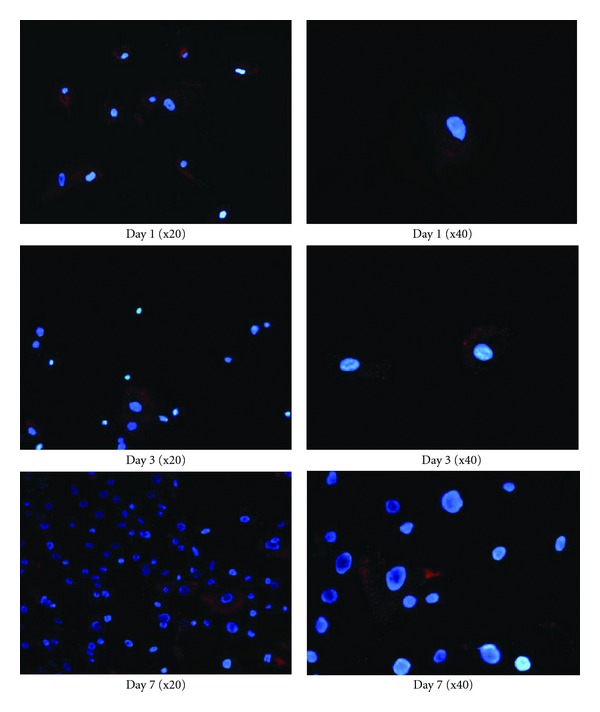
Immunofluorescence of rhodamine B (red) tagged to SPIO shows uptake of contrast agent in labeled SMC over 7 days after cell labeling.

At the incubation concentrations illustrated in Figure 6, namely, [Gd_2_O_3_] = 0.02 mM and [SPIO] = 0.0036 mM, *T*_1_- and *T*_2_-relaxation times measured in the mixed EC and SMC pellets were averages between those found in either cell type alone (Figure 7), as expected. At these concentrations, a vast difference in *T*_2_ values exists, which provides the means to achieve differential image contrast shown in Figure 6. Measured *T*_1_ and *T*_2_ values were relatively stable over 1 to 7 days post-cell labeling (Figure 7), except for a trend towards increased *T*_2_ relaxation times in SPIO-labeled cells. Immunofluorescence reveals that the SPIO is retained in fewer cells at days 3 and 7 compared to day 1, where virtually all cells were labeled (Figure 8). This suggests that in addition to the consequence of cell proliferation, contrast leakage from cells may have been present, which would account partly for increased T2 with time.

## 4. Discussion

In this study, we combined Gd_2_O_3_-labeling and SPIO-labeling for simultaneous imaging of two cell populations at the same spatial location. While both are metal oxides, SPIO was used for its negative *T*_2_ contrast, whereas Gd_2_O_3_ was used for its positive *T*_1_ contrast. Our results demonstrated that negative *T*_2_ or positive *T*_1_ contrast was achieved only when SPIO- or Gd_2_O_3_-labeled cells, respectively, were present either on their own or mixed with cells labeled with the other agent. The absence of contrast change was consistent with the absence of cells labeled with the agent of interest. The concurrent dual contrast visualization of labeled human aortic endothelial and smooth muscle cells was demonstrated at 7 Tesla and persisted up to at least 7 days after cell labeling.

The ability to visualize simultaneously two cell populations is gaining interest and can significantly expand the horizons of cellular MRI applications. In the most straightforward scenario, one can distinguish cells injected at different locations. This is particularly relevant in stem cell therapy, where the migration patterns of therapeutic cells (e.g., expressing antitumor genes) need to be studied and optimized [18]. Another scenario is to track cells or labeled drug delivery vehicles injected at the same location but at different time points, such as for antivascular cancer therapy. The most complex scenario is to visualize two cell populations occupying the same spatial and temporal domain. This is the scenario for which we have demonstrated our concurrent SPIO- and Gd_2_O_3_-labeling approach, one which opens the greatest range of opportunities for studying cell-cell interactions (e.g., in cell therapies, cancer growth, vascular development, and tissue regeneration). Towards the goal of concurrent imaging, several groups have made important recent contributions. Dual contrast from SPIO and manganese oxide was demonstrated for tracking transplanted cells in the rat brain, and differential contrast was achieved when the two cell populations were injected into different sides of the brain [13]. For visualizing different targets at the same spatial location, CEST agents hold much promise, but its low sensitivity [19] remains an active area of research. All reports of CEST to date, whether used alone [15, 20] or combined with SPIO [14], remain limited to experiments in doped solutions or direct injections into tissue. Our approach of dual SPIO and Gd_2_O_3_ labeling is, to our knowledge, the first effort to combine high-sensitivity agents of opposing contrast and demonstrate feasibility in two distinct cell populations occupying the same spatial location.

In order to establish the coexistence of *T*_1_- and *T*_2_-contrast mechanisms, this study investigated the contrast agent concentrations where a dual contrast approach may be feasible. For both SPIO and Gd_2_O_3_, low concentrations were necessary to achieve the desired shift in *T*_2_ or *T*_1_ without incurring appreciable shifts in the alternate contrast. For instance, if a high concentration of Gd_2_O_3_ was employed for labeling, the resulting negative T_2_ contrast would be indistinguishable from SPIO-labeled cells. The key to achieving “positive” and “negative” contrast concurrently involves (1) maximizing the change in the relevant relaxation time and (2) setting appropriate acquisition parameters to highlight contrast differences. To achieve even greater differential contrast, we need to optimize the concentrations used beyond this initial investigation where large steps in contrast agent concentrations were evaluated. For instance, although [SPIO] = 0.0036 mM generated the desired *T*_2_ reduction, a smaller level of iron may yield an adequate *T*_2_ decrease without incurring a significant change in *T*_1_. This optimization is useful for identifying the fraction of each labeled cell type that can be detected in a mixed cell population. Optimization is also necessary when transitioning from 7 tesla (used in this study to establish a platform for preclinical and small animal imaging) to clinical field strengths (1.5 and 3.0 Tesla) where even greater differential contrast can be reaped. Application of the proposed method in vivo will likely meet with new challenges and may need to be optimized differently.

The question arises as to whether or not similar results may be achieved using SPIO and Gd chelates, a more familiar agent for cell labeling. The answer is probably no, the reason being that the Gd_2_O_3_ employed in this study is much larger than conventional Gd chelates, whose small size and high hydrophilicity [21] hinder spontaneous cell uptake and retention. In fact, labeled cells showed *T*_1_ enhancement at incubation concentrations (<0.1 mM) much lower than those (>25 mM) used in most Gd studies [11, 22], owing to efficient internalization of Gd_2_O_3_. A cellular content of 7.26–34.1 pg Gd/cell was achieved over an incubation concentration interval of 0.02–0.1 mM, which is much higher than 0.119 pg/cell [23] or 0.3 pg/cell at 25 mM incubation concentration [21] reported in other Gd studies. Also, unlike Gd chelates that show decreased contrast effect by day 3 after cell labeling [21], the Gd_2_O_3_ maintained *T*_1_ enhancement for at least 7 days, most likely due to enhanced cell retention of a larger Gd structure. Detailed investigation into the mechanism of contrast uptake, distribution within the cell, and retention duration will be necessary for optimizing this Gd_2_O_3_ prototype for biomedical uses.

Labeling multiple cell types is especially relevant to tissue engineering and regenerative medicine, where different cell types are often cocultured to grow an organ. In our work, EC and SMC were investigated as they are key components in growing blood vessels. However, in all cell-labeling applications, a few important considerations should be noted. The addition of contrast agents should not adversely affect normal cell secretions and cellular interactions. Also, to maintain distinct cell identification with time, agents should not cross from one cell type into another. This possibility is unlikely for Gd_2_O_3_, since its internalization was assisted with the use of positively charged PLL. However, for SPIO, there exists the potential for spontaneous uptake by macrophages and other phagocytic cells. Further investigation is warranted using in vitro and in vivo systems to study the impact of dual cell labeling on cell interaction and tissue growth.

## 5. Conclusions

The present study demonstrated the feasibility of using Gd_2_O_3_ and SPIO nanoparticles for obtaining simultaneous dual contrast at 7 Tesla of two different cell populations occupying the same spatial location. Even greater differential contrast is expected at clinical field strengths of 1.5 and 3.0 Tesla. The approach holds great potential for opening new opportunities in regenerative medicine, angiogenesis, stem cell therapy, and labeled drug delivery vehicles.

## Figures and Tables

**Table 1:** Cellular uptake of iron (Fe) or gadolinium (Gd) measured on ICP-AES.

Incubation medium [Fe] (mM)	Mass of Fe (pg/cell)	Incubation medium [Gd] (mM)	Mass of Gd (pg/cell)
0	0	0	0
0.0036	0.77	0.002	0.71
0.009	1.87	0.02	7.26
0.018	3.21	0.1	34.1
0.036	6.17	0.2	84.9

A linear correlation exists between Fe incubation concentration and cellular uptake (*r* = 0.9997, *P* < 1*e* − 6) and between Gd incubation concentration and cellular uptake (*r* = 0.995, *P* < 1*e* − 4).

**Table 2:** Effect of Gd_2_O_3_ incubation concentration on MR relaxation times and cell function.

Incubation medium [Gd] (mM)	Measured MR relaxation times		Cell function assays	
	*T*_ 1_ (ms)	*T*_ 2_ (ms)	Viability (%)	Proliferation (%)
0	2168 ± 161	83.5 ± 10.3	100 ± 9	100 ± 16
0.002	1920 ± 187	69.4 ± 10.5	105 ± 14	104 ± 21
0.02	1650 ± 224	50.3 ± 9.1	112 ± 18	81 ± 9
0.1	950 ± 137	15.4 ± 3.6	95 ± 21	76 ± 13
0.2	630 ± 91	12.6 ± 2.7	84 ± 3	61 ± 5^∗^

^∗^ *P* < 0.05, significantly different from control (0 mM).

**Table 3:** Effect of SPIO incubation concentration on MR relaxation times and cell function.

Incubation medium [Fe] (mM)	Measured MR relaxation times		Cell function assays	
	*T*_ 1_ (ms)	*T*_ 2_ (ms)	Viability (%)	Proliferation (%)
0	2166 ± 135	85.7 ± 9.0	100 ± 14	100 ± 18
0.0036	1263 ± 193	16.1 ± 2.6	96 ± 19	92 ± 8
0.009	828 ± 127	12.6 ± 0.6	96 ± 15	96 ± 19
0.018	613 ± 65	11.3 ± 0.6	95 ± 13	89 ± 12
0.036	630 ± 60	10.9 ± 0.5	86 ± 16	84 ± 15
